# Supplementary material for: Exploring roles of the chitinase ChiC in modulating Pseudomonas aeruginosa virulence phenotypes
Source: Microbiol Spectr. 2024 May 31;12(7):e00546-24. doi: 10.1128/spectrum.00546-24 (PMC11218509; doi:10.1128/spectrum.00546-24)
Supplement: Supplemental figures — Fig. S1 to S11. [file spectrum.00546-24-s0001.docx]

**Supplementary information**

**Exploring roles of the chitinase ChiC in modulating *Pseudomonas aeruginosa* virulence phenotypes**

**Per Kristian Thorén Edvardsen^1*^, Fatemeh Askarian^2^, Raymond Zurich^2^, Victor Nizet^2,3^, and Gustav Vaaje-Kolstad^1*^**

**^1^Faculty of Chemistry, Biotechnology and Food Science, Norwegian University of Life Sciences, Ås, Norway.**

**^2^Division of Host-Microbe Systems & Therapeutics, Department of Pediatrics, UC San Diego School of Medicine, La Jolla, CA, USA**

**^3^Skaggs School of Pharmacy and Pharmaceutical Sciences, UC San Diego, La Jolla, CA, USA.**

***Correspondence:**

**Per Kristian Thorén Edvardsen**

[**per.kristian.edvardsen@nmbu.no**](mailto:per.kristian.edvardsen@nmbu.no)

**Gustav Vaaje-Kolstad**

[**gustav.vaaje-kolstad@nmbu.no**](mailto:gustav.vaaje-kolstad@nmbu.no)

**Keywords: *Pseudomonas aeruginosa*, chitinase, GH18, virulence, biofilm.**


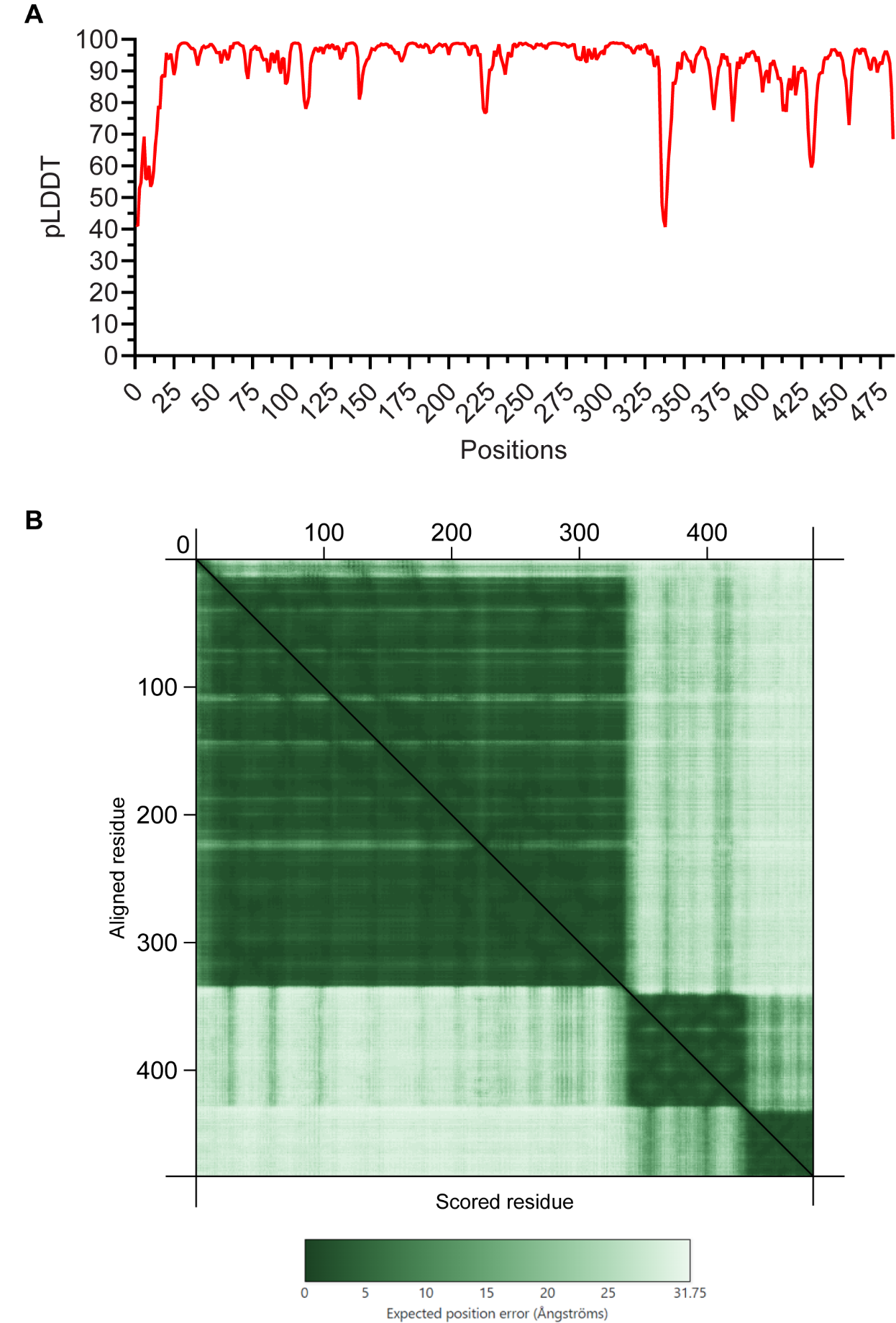


**Supplementary figure 1**. Alphafold2 structure prediction statistics for the predicted Alphafold2 model of ChiC. **(A)** Per-residue pLDDT score plot with an average pLDDT score of 92,21, and **(B)** the predicted aligned error for the model.


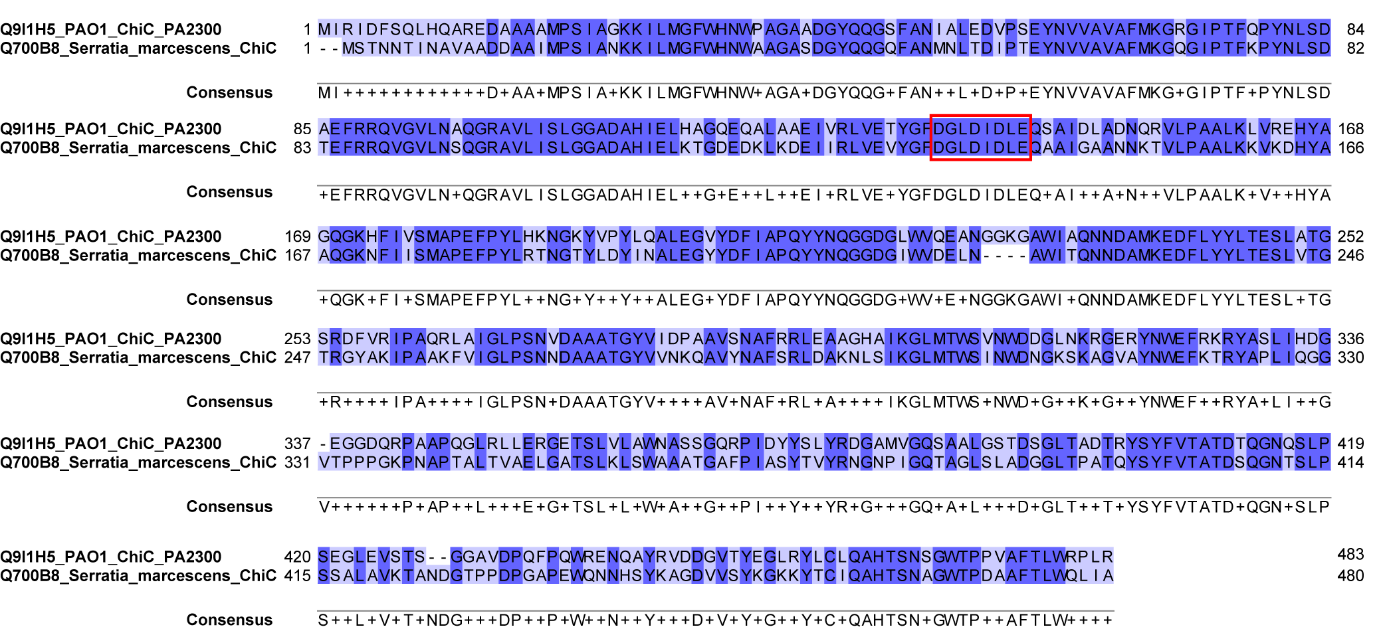


**Supplementary figure 2**. Sequence alignment of ChiC and *Sm*ChiC using MUSCLE with default settings in Jalview (1). The conserved DXXDXDXE motif is marked with a red square.


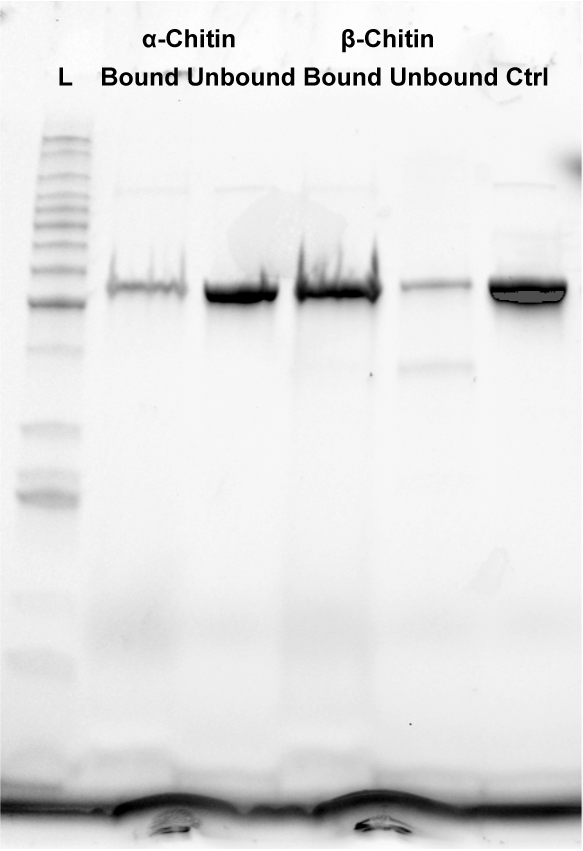


**Supplementary figure 3**. ChiC binding to α-chitin and β-chitin. SDS-PAGE analysis showing the bound and unbound fractions of ChiC to α- and β-chitin, including a control with only ChiC. BenchMark™ Protein Ladder (Thermo Fisher Scientific) was used as the ladder (L).


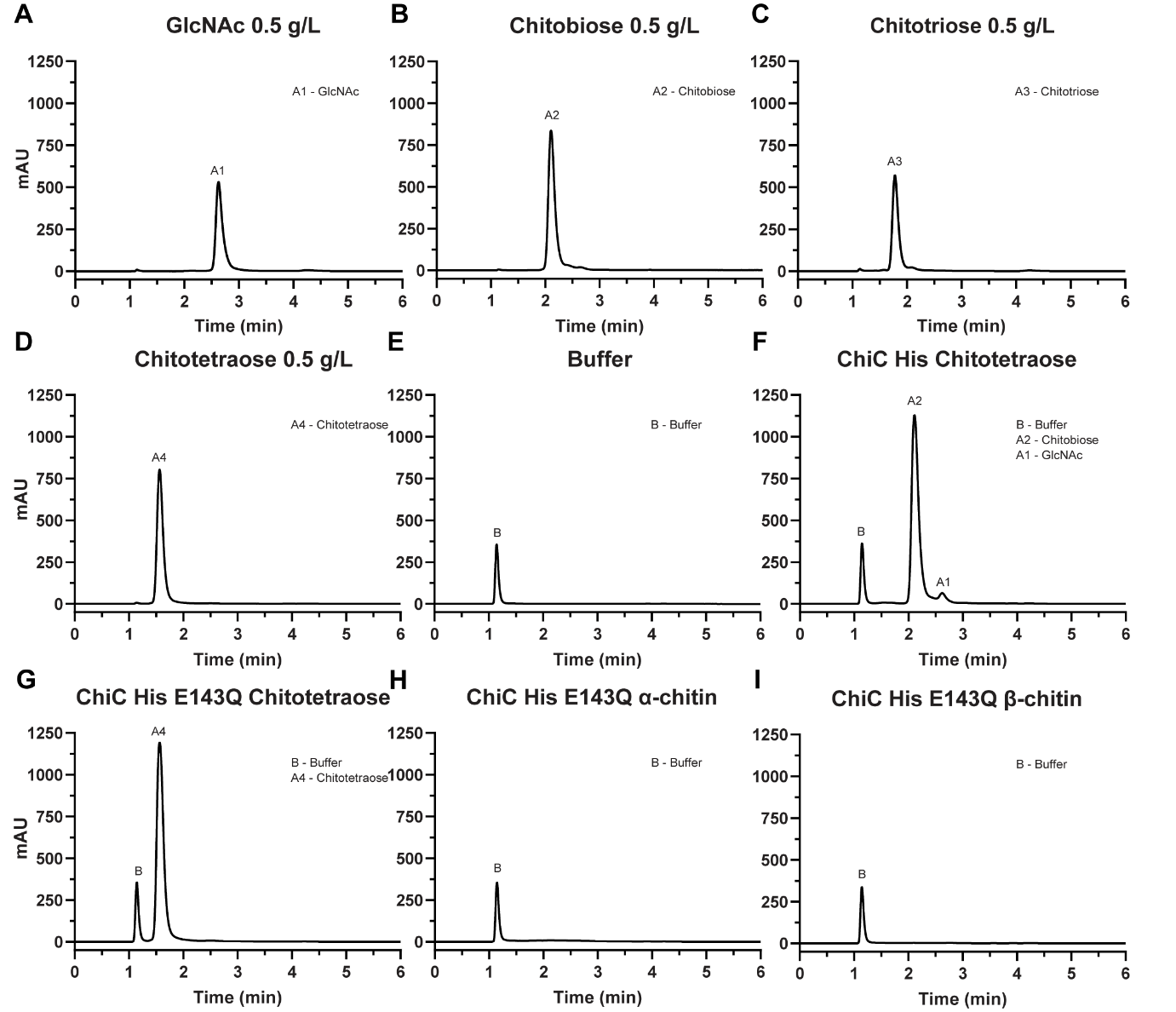


**Supplementary figure 4**. Chromatograms from the Rezex analytical column showing the different profiles of the sugar standards and select enzymatic reactions with different substrates. The chromatogram profile of (A) 0.5 g/L GlcNAc, (B) 0.5 g/L (GlcNAc)_2_/chitobiose, (C) 0.5 g/L (GlcNAc)_3_/chitotriose, (D) 0.5 g/L (GlcNAc)_3_/chitotetraose, (E) Buffer control (Tris-HCl pH 7.5), (F) ChiC His incubated with (GlcNAc)_4_/chitotetraose, (G) ChiC_E143Q_ incubated with (GlcNAc)_4_/chitotetraose, (H) ChiC_E143Q_ incubated with β-chitin and (I) ChiC_E143Q_ incubated with β-chitin. Absorbance at wavelength 194 nm.


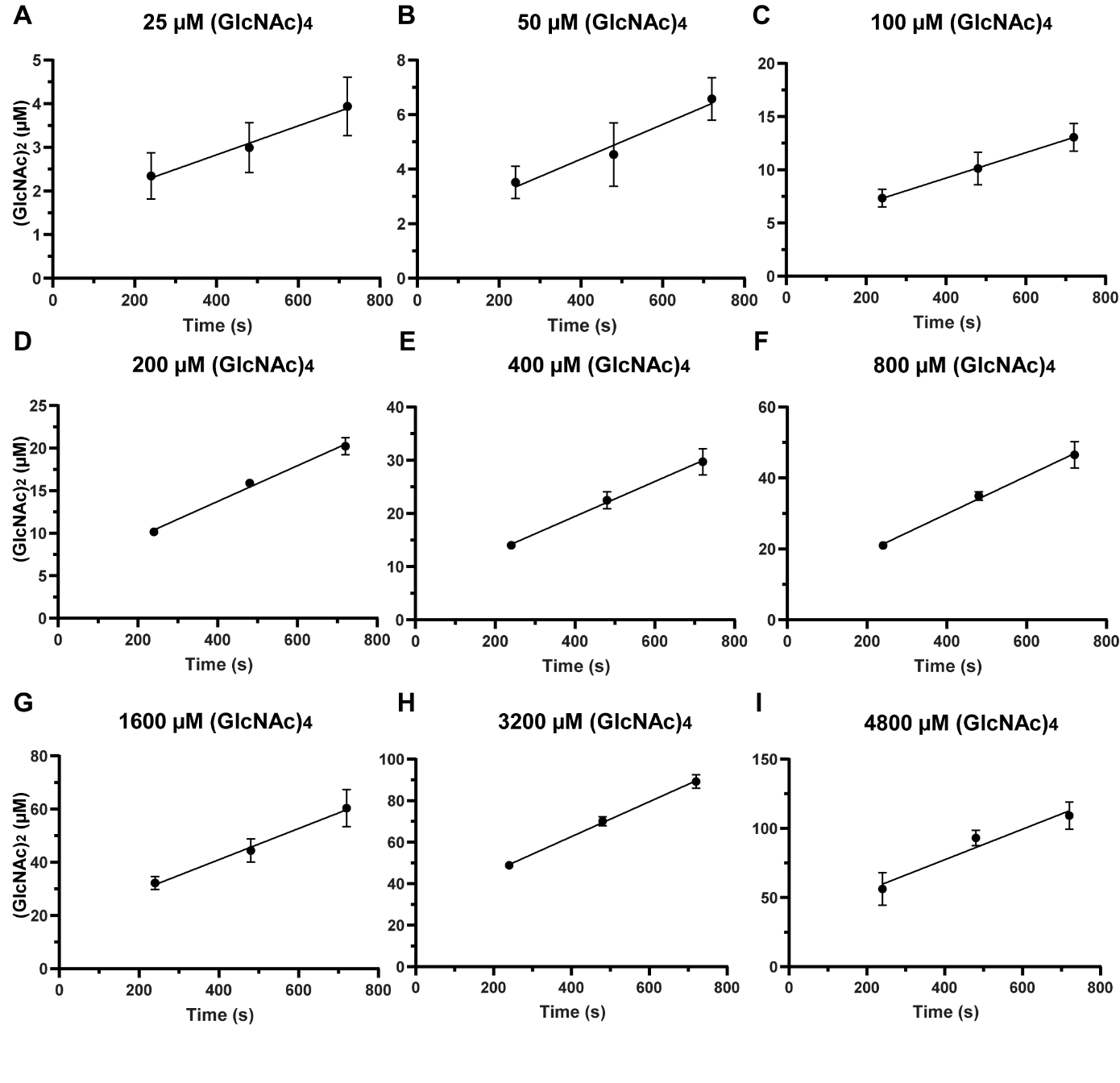


**Supplementary figure 5**. Determination of initial velocities for Michaelis-Menten kinetic analysis. The amount of (GlcNAc)_2_ formed by ChiC (in µM) was analyzed using varying concentrations of (GlcNAc)_4_ (A) 25 µM, (B) 50 µM, (C) 100 µM, (D) 200 µM, (E) 400 µM, (F) 800 µM, (G) 1600 µM, (H) 3200 µM, and (I) 4800 µM.


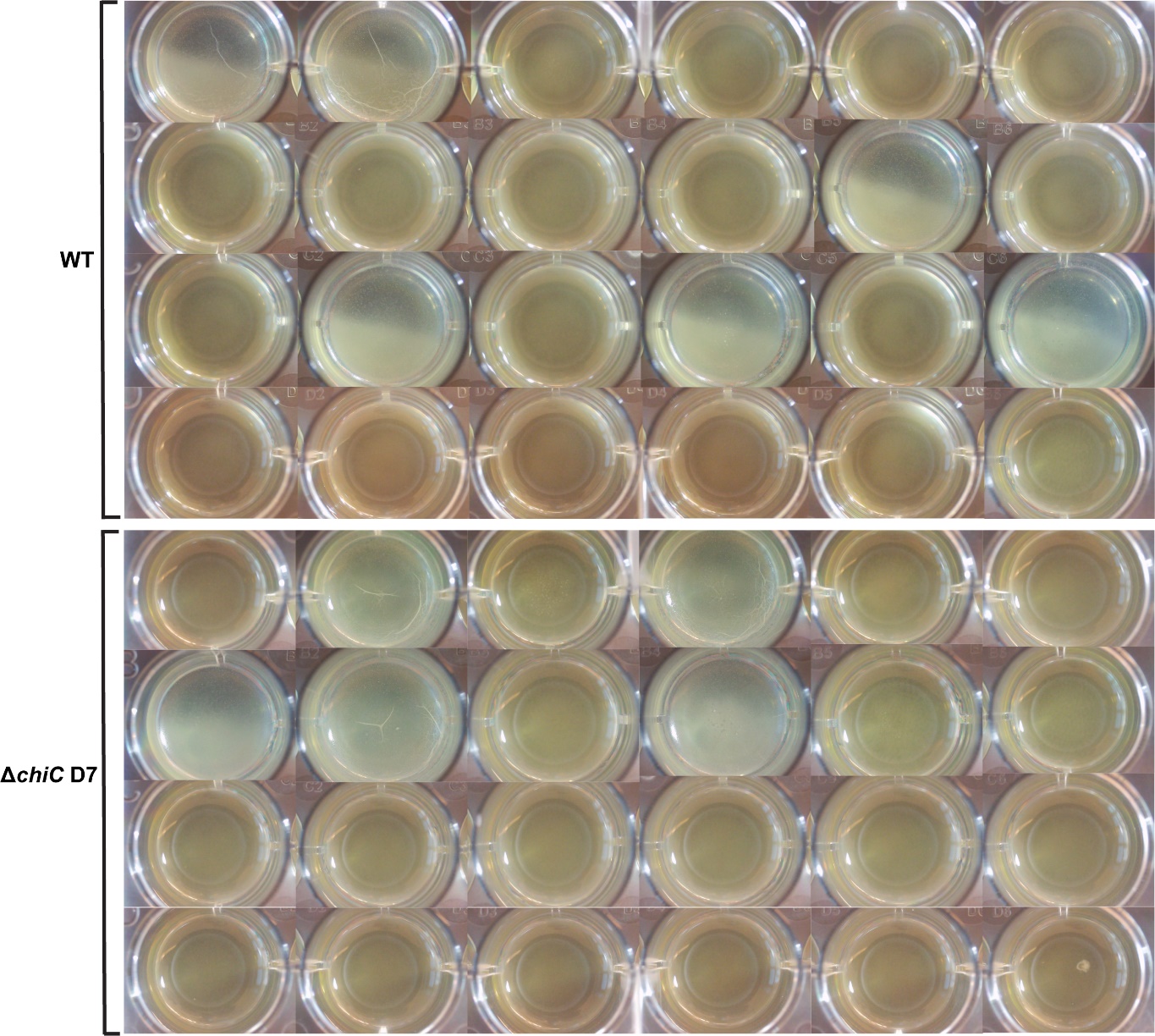


**Supplementary figure 6**. Pellicle formation in PA WT (PA14) and PA *chiC*::MAR2xT7 D7 mutant at 20 ℃. Bacteria were grown in 1 mL of T-broth for one week statically in a 24-well plate. Pellicles formed by the bacteria in each well were photographed from above.


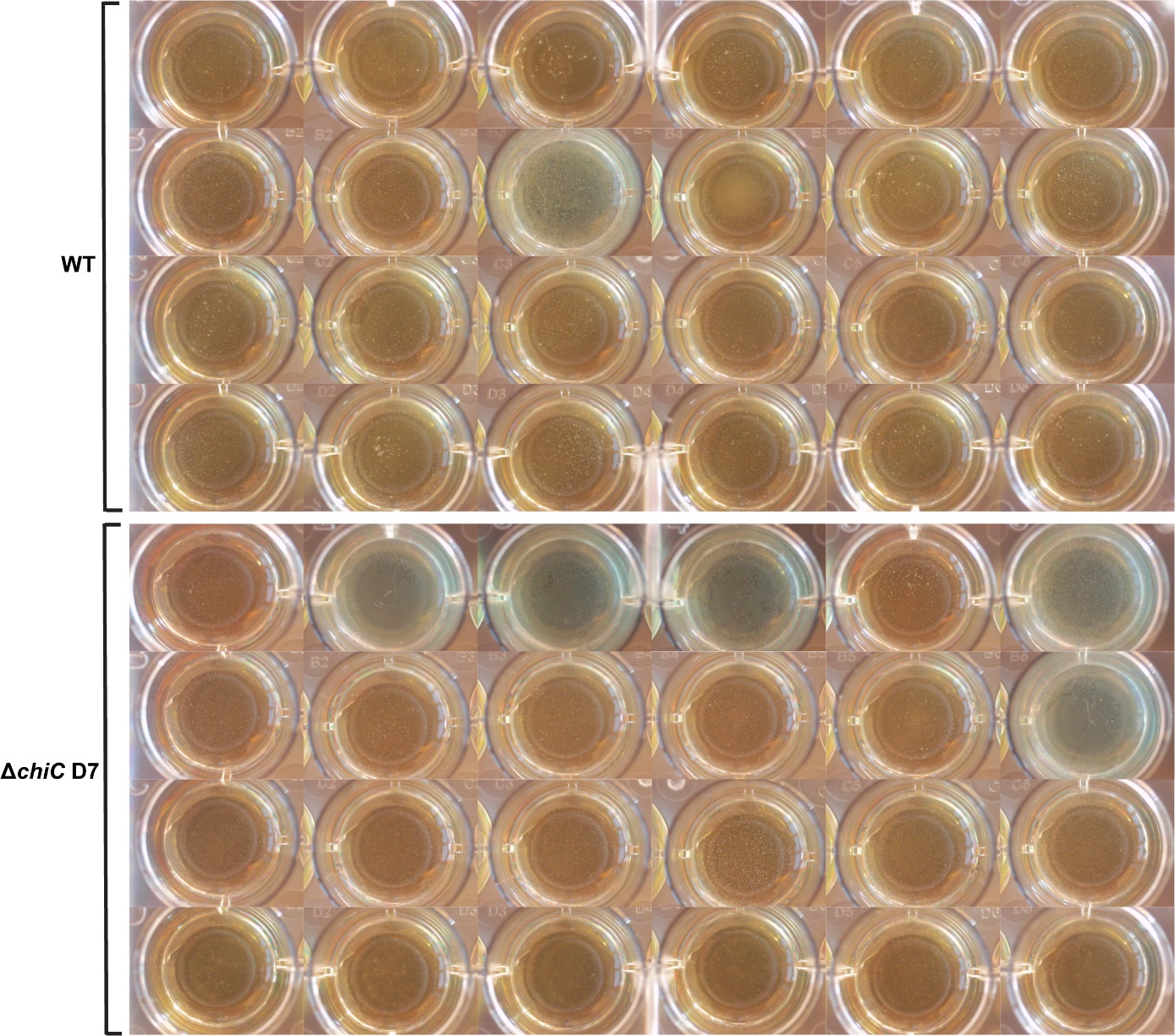


**Supplementary figure 7**. Pellicle formation in PA WT (PA14) and PA *chiC*::MAR2xT7 D7 mutant at 37 ℃. Bacteria were grown in 1 mL of T-broth for one week statically in a 24-well plate. Pellicles formed by the bacteria in each well were photographed from above.


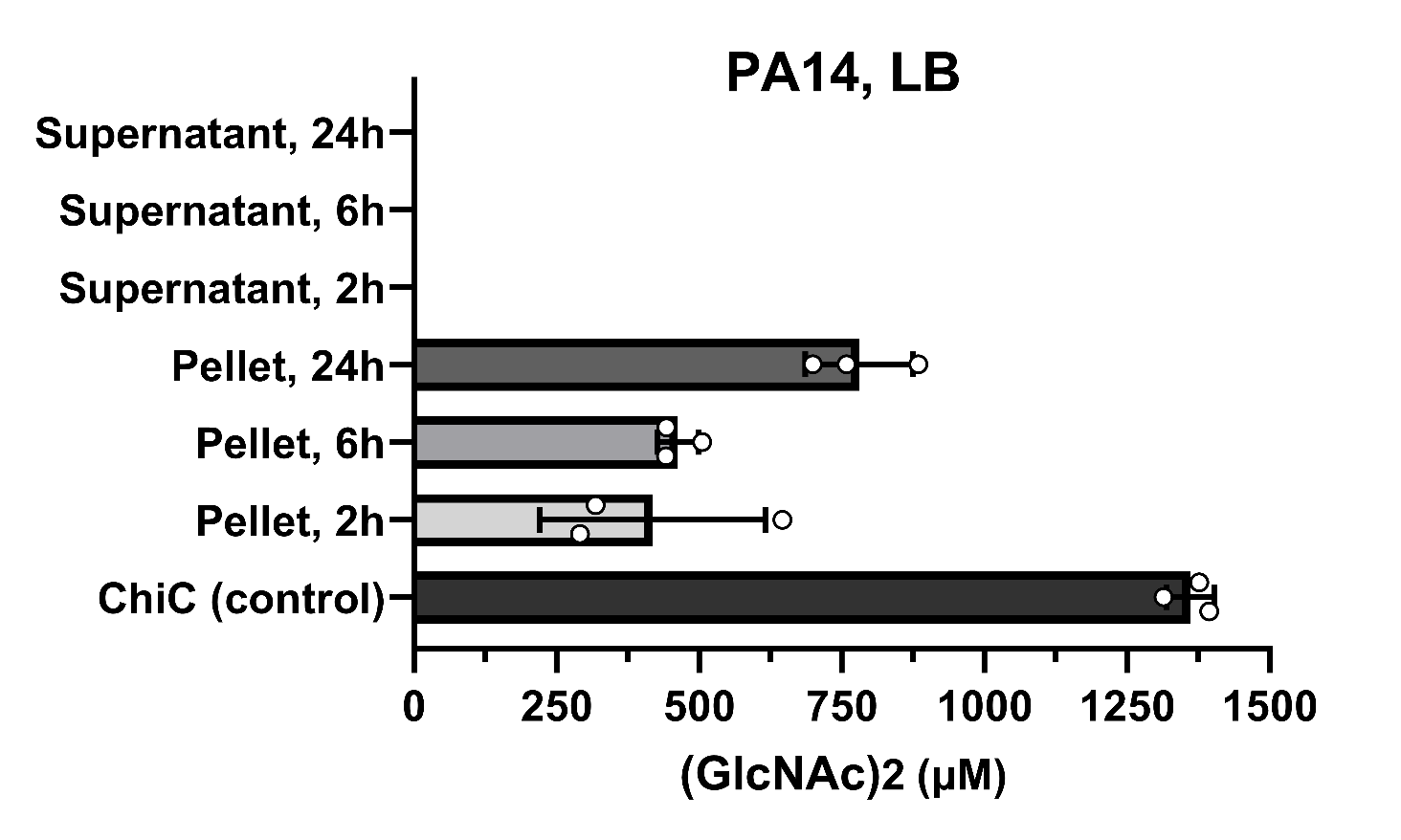
**Supplementary figure 8**. Quantification of chitinase activity of PA (PA14) at different growth phases in LB medium. Activity is represented by the concentration of generated (GlcNAc)_2_ (µM) by the bacteria and the cell-free supernatants upon incubation with (GlcNAc)_4_ for 2 hours after the given growth phase was reached. Purified ChiC (1 µM) was used as a positive control.


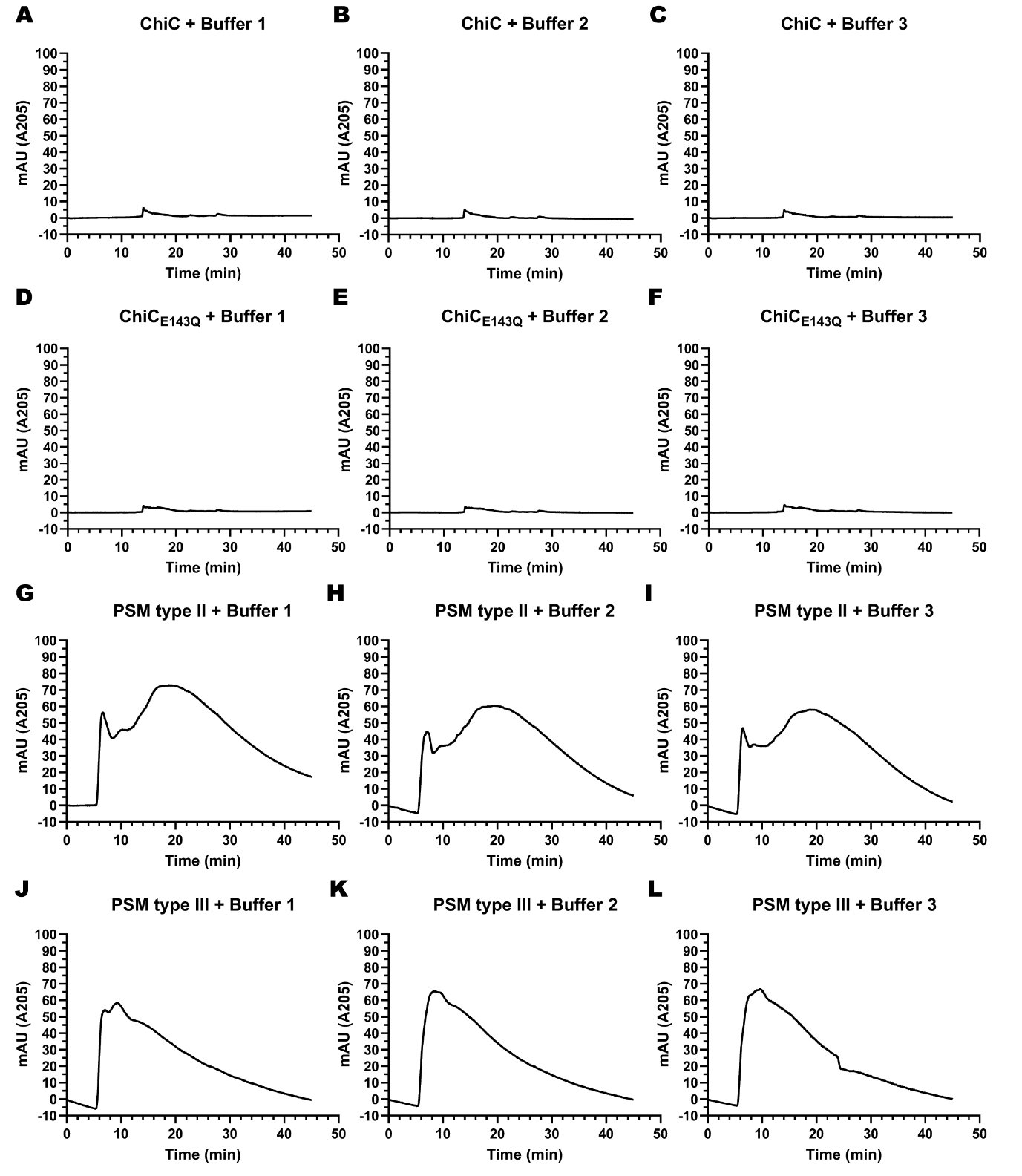


**Supplementary figure 9**. Chromatograms from the SEC analytical column showing different elution profiles for the different control reactions of ChiC and porcine stomach mucin (PSM). Each control reaction was run in triplicate with (A-C) showing the chromatograms of ChiC incubated with buffer, (D-F) showing the chromatograms of ChiC_E143Q_ with buffer, (G-I) showing the mucin extract type II incubated with buffer and (J-L) showing the mucin extract type III incubated with buffer. Absorbance was measured at 205 nm.


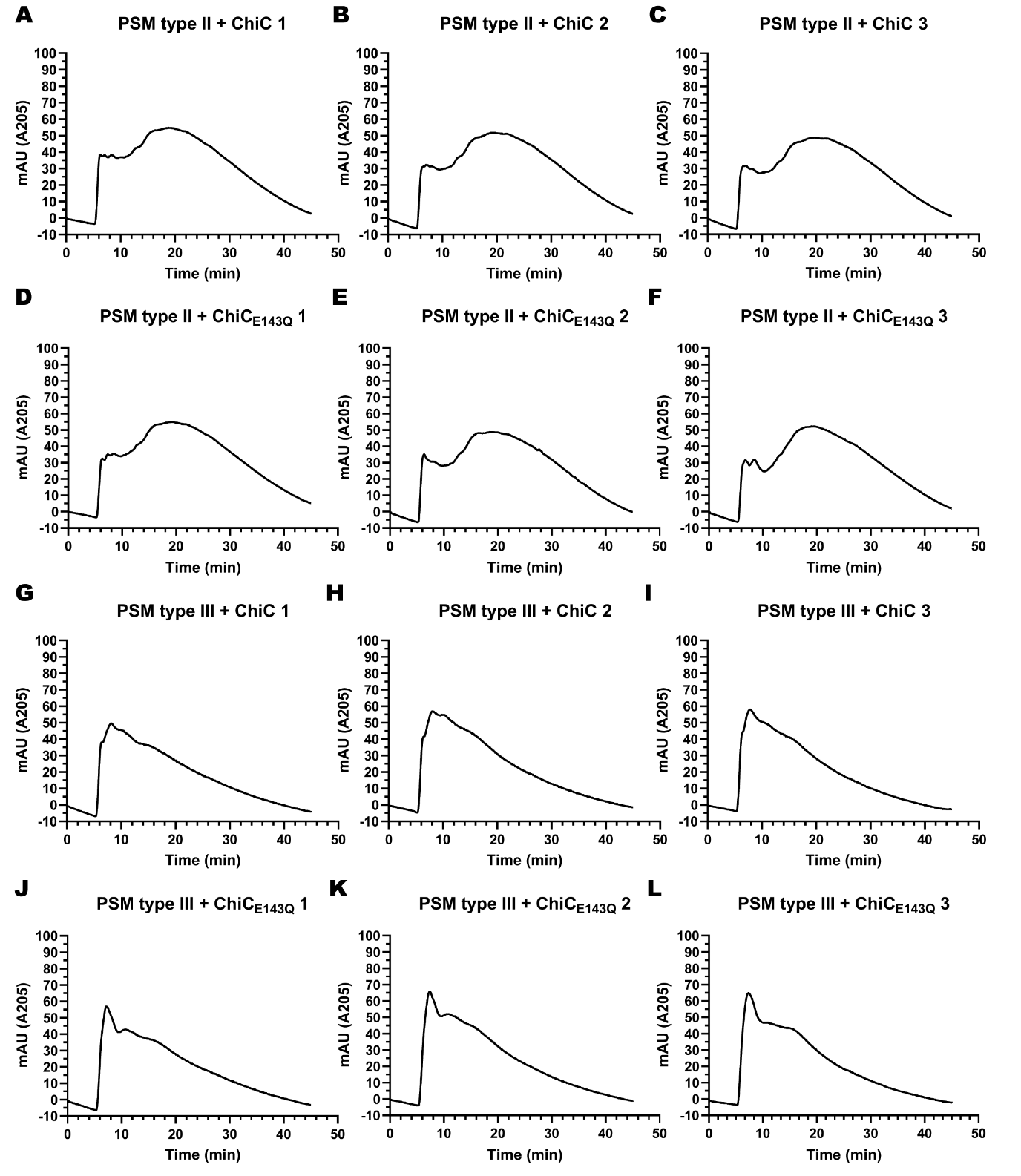


**Supplementary figure 10**. Chromatograms from the SEC analytical column showing different elution profiles for the different reactions of ChiC and ChiC_E143Q_ incubated with porcine stomach mucin (PSM). Each reaction was run in triplicate with (A-C) showing the chromatograms of ChiC incubated with mucin extract type II, (D-F) showing the chromatograms of ChiC_E143Q_ with mucin extract type II, (G-I) showing the chromatograms of ChiC incubated with mucin extract type III and (J-L) showing the chromatograms of ChiC_E143Q_ with mucin extract type III. Absorbance was measured at 205 nm.


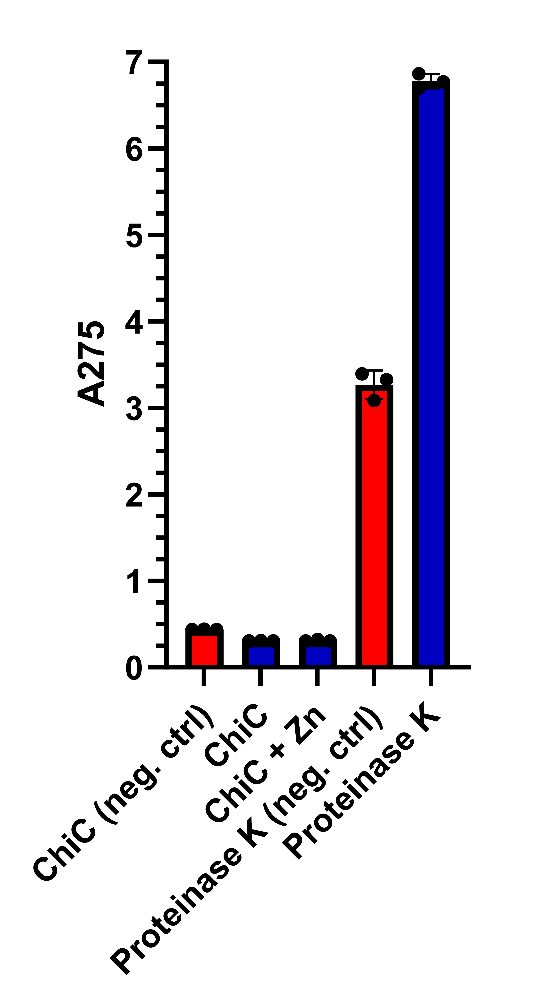


**Supplementary figure 11**. Measurement of protease activity of ChiC and Proteinase K against casein. The data plotted is represented as the mean ± SD of three replicates. Absorbance was measured at 275 nm.

1. Waterhouse AM, Procter JB, Martin DMA, Clamp M, Barton GJ. 2009. Jalview Version 2 - A multiple sequence alignment editor and analysis workbench. Bioinformatics 25:1189–1191.
